# 154. Prevalence and Antimicrobial Resistance of *Enterococcus* species in a Tertiary Center Children’s Hospital in Korea

**DOI:** 10.1093/ofid/ofac492.232

**Published:** 2022-12-15

**Authors:** Hyejin So, Jina Lee

**Affiliations:** Chungnam National University Sejong Hospital, Sejong, Ch'ungch'ong-namdo, Republic of Korea; Asan Medical Center, University of Ulsan College of Medicine, Seoul, Kyonggi-do, Republic of Korea

## Abstract

**Background:**

Increasing prevalence of *Enterococcus* spp. as a nosocomial pathogen with high-level resistance to multiple antimicrobials has been a challenge throughout the years, and now, even to the pediatric age. This study is a pioneer research which was designed to determine the prevalence of different antibiogram patterns of enterococcal strains in the pediatric age.

**Methods:**

In this retrospective review, we included *Enterococcus* species isolated from normal body fluid including blood and CSF from March 2014 through September 2020 at Asan Medical Center Children`s Hospital. Electronic Medical Records (EMR) was reviewed for clinical and demographic data.

**Results:**

A total of 205 different clinical strains of *Enterococcus* species were collected. Isolated strains were *E. faecalis* (47.3%), *E. faecium* (41.5%), *E. avium* (4.9%), *E. gallinarum* (2.9%), *E. raffinosus* (2.0%) and others (1.5%). No strains were resistant to linezolid. Vancomycin or teicoplanin resistant strains were 26.3% (vancomycin 17.1%, teicoplanin 9.3%), most of which were *E. faecium* (vancomycin 13.7%, teicoplanin 9.3%). Ampicillin or penicillin resistant strains were 82.9% (ampicillin 39.0%, penicillin 43.9%), most of which were also *E. faecium* (ampicillin 35.1%, penicillin 36.1%). High-level streptomycin or gentamicin resistant strains were 46.8% (streptomycin 13.2%, gentamicin 33.7%), mostly *E. faecalis* (streptomycin 7.8%, gentamicin 19.0%). The prevalence of antimicrobial resistance differed significantly between *E. faecalis* and *E. faecium*; *E. faecalis* being nearly all susceptible to ampicillin and penicillin except 6 strains which was an interesting finding because ampicillin-susceptible, penicillin-resistant *E. faecalis* strains were found.

Antibiotic Resistance Pattern of Enterococcal Strains

**Figure:**
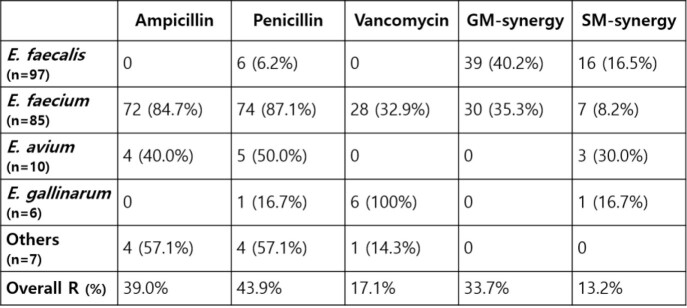

MIC of ASPR Enterococcus species by Etest

**Figure:**
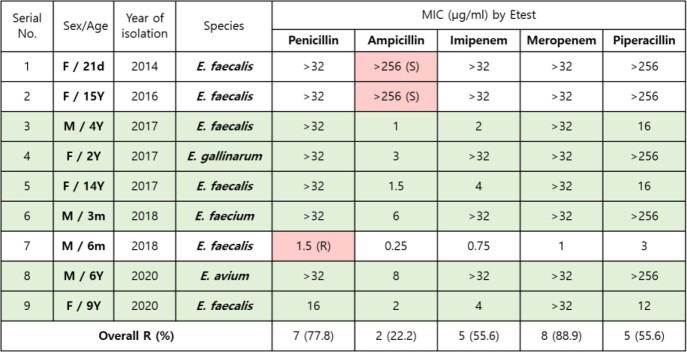

**Conclusion:**

Consistent findings with previous studies based on adults are that *E. faecalis* and *E. faecium* were the predominant strains causing infection in children as well. There are reports about currently emerging ampicillin-susceptible but penicillin-resistant(ASPR) *E. faecalis* strains which were also found in this study and so therefore further molecular studies about this newly emerging ASPR *E. faecalis* is in progress.

**Disclosures:**

**All Authors**: No reported disclosures.

